# To wait or not to wait: Optimal time interval between the first and second blood-culture sets to maximize blood-culture yield

**DOI:** 10.1017/ash.2022.27

**Published:** 2022-03-25

**Authors:** Valeria Fabre, George F. Jones, Yea-Jen Hsu, Karen C. Carroll, Sara E. Cosgrove, for the CDC Prevention Epicenters Program

**Affiliations:** 1 Division of Infectious Diseases, Department of Medicine, Johns Hopkins University School of Medicine, Baltimore, Maryland; 2 Department of Health Policy and Management, Johns Hopkins Bloomberg of School of Public Health, Baltimore, Maryland; 3 Division of Clinical Microbiology, Department of Pathology, Johns Hopkins University School of Medicine, Baltimore, Maryland

## Abstract

The optimal timing of blood culture (BCx) sets collection has not been evaluated with continuous BCx detection systems. The yield of BCx was similar between short intervals (median, 3 minutes) and longer intervals (median, 16 or 43 minutes) among 5,856 BCx, except for improved polymicrobial bacteremia detection with long-interval BCx.

Blood culture (BCx) stewardship is important to ensure appropriate diagnosis of bloodstream infections, and it includes optimization of BCx collection.^1^ Although studies have addressed the impact detection of the timing from BCx collection to incubation or in relation to fever on bacteremia,^2–4^ data on the optimal interval between successive BCx sets are very limited. Historically, a wait time between BCx sets has been recommended out of concern that simultaneous BCx may miss intermitent bacteremias or bacteremias with low bacterial concentration.^5,6^ However, a study evaluating 7,783 BCx processed with the BACTEC 660 infrared detection system in 1994 showed that BCx positivity was not increased by lengthening intervals between BCx in a 24-hour period.^7^ The American Society for Microbiology and the Clinical and Laboratory Standards Institute recommend collecting multiple sets simultaneously or over a short time, except when documentation of continuous bacteremia is required for patients with endovascular infection.^8^ The Infectious Diseases Society of America guidelines recommend sequential BCx over minutes in urgent situations when prompt antibiotic initiation is needed (eg, septic shock) and longer intervals (several hours or more) for nonurgent situations. A reference for this recommendation is not provided.^9^ Furthermore, whether simultaneous BCx are widely accepted in clinical practice remains unlcear.

We evaluated the impact of timing on BCx yield in the era of continuous BCx detection instruments.

## Methods

### Study population

We retrospectively evaluated the first 2 sets of BCx obtained within the first 24 hours from patients ≥18 years of age presenting to the emergency department at The Johns Hopkins Hospital (JHH) between January 28, 2017, and October 31, 2019. JHH is an academic hospital in Baltimore, Maryland. We excluded patients with additional BCx obtained beyond the first 2 sets to avoid bias associated with increased likelihood of bacteremia detection with additional sets,^10^ solitary BCx (ie, 1 BCx set in a 24-hour period), and BCx growing contaminants.

BCx were processed in the JHH Microbiology Clinical Laboratory using the BD Bactec FX BCx system (Becton Dickinson, Sparks, MD) for organism detection. BCx bottles were incubated for 5 days, and identification of microorganisms was performed using matrix-assisted laser desorption ionization-time of flight mass spectrometry (MALDI-TOF MS) and the Verigene BCID-GP panel. BCx collection times and results were electronically extracted from the electronic health record (EHR). The recommended volume for BCx is 10 mL per bottle for adults.

### Definitions

BCx contamination was defined using a standard laboratory definition (growth in a single BCx set of any of the following organisms: *Bacillus* spp, coagulase-negative staphylococci, *Corynebacterium* spp, *Micrococcus* spp, *Cutibacterium acnes*, and viridans group streptococci). A BCx pair refers to the first 2 sets of BCxs drawn from a patient (1 set includes 1 aerobic and 1 anaerobic bottle). BCx positivity was defined as the proportion of BCx pairs with growth of a noncontaminant organism in 1 or both BCx sets. BCx with collection times in Epic software (Epic, Verona, WI) separated by 0–9 minutes were defined as short-interval or simultaneous blood cultures, and sets separated by ≥10 minutes (10–29 minutes or ≥30 minutes) were defined as long-interval BCx.

### Statistical analysis

BCx positivity was compared across intervals and were stratified by causative organism. Analyses utilized χ^2^ tests or Fisher exact tests when appropriate (first *P* value reported). Nonparametric tests for trend were used to further evaluate the impact of periodicity on BCx positivity. We accounted for potential differences in BCx practices in time through a regression analysis. A 2-sided *P* value <.05 was considered statistically significant. Analyses were performed using Stata software (2019, StataCorp, College Station, TX).

This study was approved by the Johns Hopkins Medicine Institutional Review Board.

## Results

Of 6,938 BCx identified within the study period, 1,082 (15.6%) were excluded: 857 due to single BCx sets, 161 due to ≥3 BCx sets in 24 hours, and 64 due to BCx contaminants. Of the 5,856 BCx sets included in the analysis, or 2,928 pairs, 789 (27%) of 2,928 pairs were drawn 0–9 minutes apart (median, 3 minutes; interquartile range [IQR], 0–8); 1,733 (59%) were drawn 10–29 minutes apart (median, 16 minutes; IQR, 13–21); and 406 (14%) were drawn with a ≥30-minute interval (median 43 minutes; IQR, 34–80). The proportion of BCx drawn 0–9 minutes increased from 23.5% in 2017 to 36% in 2018 to 40% in 2019 (*P* < .01 for all comparisons).

The overall BCx positivity rate was 15% (439 of 2,928); most cases were monomicrobial, and 57 (13%) of these 439 cases were polymicrobial. BCx positivity rates were similar among the 3 collection time groups after adjusting for month or year of BCx collection: 13% (105 of 789) for 0–9 minutes, 15% (265 of 1,733) for 10–29 minutes, and 17% (69 of 406) for ≥30 minutes (*P* = .20, test for trend *P* = .07) (Table 1) (Supplementary Material). In a subgroup analysis excluding 274 BCx pairs with antibiotics given before the first or second BCx set, rates remained similar (14%, 16%, and 15% positivity for the 3 groups, respectively; *P* = .40; test for trend *P* = .27) (Supplementary Material).

**Table 1.** Blood Culture (BCx) Positivity by Time Interval and by Causative Pathogen

Blood Culture Pairs by Interval (n = 2,928, %)	Overall Blood Culture Positivity, (n = 439, 15%)	*P* Value^a^	Positivity, Gram Positives (n = 234, 8%)	*P* Value^a^	Positivity, Gram Negatives (n = 205, 7%)	*P* Value^a^	Positivity Anaerobes (n = 24, 1%)	*P* Value^a^	Positivity Yeast (n = 7, 0.2%)	*P* Value*	Positivity Polymicrobial (n = 57, 13%)	*P* Value^a^
0–9 min (n = 789, 26.9%)	105 (13.3)	.20 .07	57 (7.2)	.51 .24	46 (5.8)	.31 .25	5 (0.6)	.12 .09	0 (0)	.04 .01	10/(9.5)	.10 .04
10–29 min (n = 1,733, 59.2%)	265 (15.3)		140 (8.1)		130 (7.5)		12 (0.7)		4 (0.2)		33 (12.5)	
≥30 min (n = 406, 13.9%	69 (17.0)		37 (9.1)		29 (7.1)		7 (1.7)		3 (0.7)		14 (20)	

a First *P* values are from χ^2^ or Fisher exact test; second *P* values are from nonparametric tests for trend.

We detected no differences in BCx positivity among short- or longer-interval BCx that grew gram-positive organisms, gram-negative organisms, or anaerobes, although we detected a non-significant trend toward increased yield for anaerobes among BCx obtained ≥30 minutes apart: 0.6% for 0–9 minutes, 0.7% for 10–29 minutes and 1.7% for ≥30 minutes (*P* = .12; test for trend *P* = .09). We also detected a trend toward increased yield for polymicrobial bacteremias for longer-interval BCx: 10 (9.5%) of 105 for 0–9 minutes; 33 (12%) of 265 for 10–29 minutes; and 14 (20%) of 69 for ≥30 minutes (*P* = .10; test for trend *P* = .04). For yeast, we also detected a trend toward increased yield: 0 (0%) for 0–9 minutes; 4 (0.2%) of 1,733 for 10–29 minutes; and 3 (0.7%) of 406 for ≥30 minutes (*P* = .04; test for trend *P* = .01).

## Discussion

We investigated the impact of time between the first and second BCx sets on BCx yield among 5,856 BCx from adult patients using a continuous BCx detection system. We found similar yield for common bacterial pathogens when sets were collected within a short interval (0–9 minutes) or a longer interval (≥10 minutes).

This study had several limitations. The interval between BCx sets derived from the EHR depends on when the individual drawing the BCx scanned the specimen into the EHR. However, we did not expect the records to be biased in one direction or the other, and simultaneous BCx are acceptable at our institution. We did not have data on blood volume per individual bottle, which may have affected bacteremia detection, because the latter is measured in aggregate using instrument software. However, we included a large number of BCx, which would have mitigated any potential disparities in blood volume in the different time groups. Also, we adjusted for potential differences in BCx practices in time through regression analysis. The importance of timing between BCx sets for polymicrobial and yeast bloodstream infections warrants further investigation in larger studies. Finally, difficult-to-grow organisms were not well represented in our cohort, and additional study is warranted to understand the impact of timing in these scenarios.

In summary, our data indicate that the timing of the second BCx set does not impact the yield of BCx to detect most common bacterial pathogens. Collecting BCx simultaneously may help streamline the clinical workflow. Future larger studies including source of infection or patient characteristics could help determine whether longer intervals improve detection of polymicrobial and yeast bloodstream infections and which patients would benefit from this practice.

## Supplementary material

For supplementary material accompanying this paper visit https://doi.org/10.1017/ash.2022.27. click here to view supplementary material
