# Supplementary material for: To wait or not to wait: Optimal time interval between the first and second blood-culture sets to maximize blood-culture yield
Source: Antimicrob Steward Healthc Epidemiol. 2022 Mar 25;2(1):e51. doi: 10.1017/ash.2022.27 (PMC9614788; doi:10.1017/ash.2022.27)
Supplement: Supplementary file 1 [file S2732494X22000274sup001.docx]

**Supplemental Material**

**Supplemental Table 1:** Subgroup analysis of blood culture positivity by time interval and by causative pathogen after excluding blood cultures with receipt of antibiotics prior to the first or second blood culture.

| **Blood culture pairs by interval**  **n=2,654 (%)** | **Overall blood culture**  **positivity**  **n=400 (15)** | ***P* value*** | **Positivity Gram positives**  **n=218 (8)** | ***P* value*** | **Positivity Gram negatives**  **n=189 (7)** | ***P* value*** | **Positivity anaerobes**  **n=19 (0.7)** | ***P* value*** | **Positivity yeast**  **n=3 (0.3)** | ***P* value*** | **Positivity polymicrobial n= 52 (13)** | ***P* value*** |
| --- | --- | --- | --- | --- | --- | --- | --- | --- | --- | --- | --- | --- |
| 0-9 min., n=723 (27.2) | 98 (13.6) | 0.40  0.27 | 54 (7.5) | 0.60  0.31 | 43 (6) | 0.25  0.42 | 4 (0.6) | 0.46  0.29 | 0 (0) | 0.70  0.68 | 10 (10) | 0.29  0.14 |
| 10-29 min., n=1,595 (60.1) | 250 (15.7) |  | 133 (8.3) |  | 124 (7.8) |  | 11 (0.7) |  | 3 (0.2) |  | 32 (13) |  |
| ≥30 min., n=336 (12.7) | 52 (15.5) |  | 31 (9.2) |  | 22 (6) |  | 4 (1.2) |  | 0 (0) |  | 10 (19) |  |

*First *P* values are from chi-square or Fisher’s exact test; second *P* values are from non-parametric tests for trend.

**Supplemental Table 2:** Basic demographic characteristics of the cohort by time interval. Short interval

(0 - 9 minutes), long interval (> 9 minutes).

| **Characteristic** | **Short interval**  **N=789 (%)** | **Long interval**  **N=2,139 (%)** | ***P* value** |
| --- | --- | --- | --- |
| **Gender** | | | |
| Female | 356 (45) | 1,064 (50) | 0.026 |
| Male | 433 (55) | 1,075 (50) |  |
| **Age (years)** | | | |
| 18-44 | 239 (30) | 694 (32) | 0.165 |
| 45-64 | 297 (38) | 828 (39) |  |
| ≥65 | 253 (32) | 617 (29) |  |

**Supplemental Table 3:** Regression analysis considering month or year of blood culture collection.

| **Model 1: By Month** | | | |
| --- | --- | --- | --- |
|  | OR | 95% CI | P value |
| Time interval |  |  |  |
| 0-9 | 1 |  |  |
| 10-29 | 1.15 | (0.90 - 1.47) | 0.267 |
| 30+ | 1.30 | (0.93 - 1.81) | 0.123 |
| Gender |  |  |  |
| Female |  |  |  |
| Male | 1.10 | (0.90 - 1.35) | 0.341 |
| Secular trend (month) | 0.99 | (0.98 - 1.00) | 0.092 |
| Constant | 0.18 | (0.13 - 0.24) | <0.001 |
| **Model 2: By Year** | | | |
|  | OR | 95% CI | P value |
| Time interval |  |  |  |
| 0-9 | 1 |  |  |
| 10-29 | 1.16 | (0.91 - 1.48) | 0.242 |
| 30+ | 1.32 | (0.94 - 1.84) | 0.104 |
| Gender |  |  |  |
| Female |  |  |  |
| Male | 1.10 | (0.90 - 1.35) | 0.354 |
| Secular trend (year) | 0.93 | (0.81 - 1.05) | 0.247 |
| Constant | 0.17 | (0.12 - 0.25) | <0.001 |
